# Evaluation of alpaca tracheal explants as an ex vivo model for the study of Middle East respiratory syndrome coronavirus (MERS-CoV) infection

**DOI:** 10.1186/s13567-022-01084-3

**Published:** 2022-09-02

**Authors:** Nigeer Te, Jordi Rodon, Rhea Creve, Mónica Pérez, Joaquim Segalés, Júlia Vergara-Alert, Albert Bensaid

**Affiliations:** 1 Unitat Mixta d’investigació IRTA-UAB en Sanitat Animal, Centre de Recerca en Sanitat Animal (CReSA), Campus de la Universitat Autònoma de Barcelona (UAB), 08193 Bellaterra, Catalonia Spain; 2 IRTA, Programa de Sanitat Animal, Centre de Recerca en Sanitat Animal (CReSA), Campus de la Universitat Autònoma de Barcelona (UAB), 08193 Bellaterra, Catalonia Spain; 3 Departament de Sanitat i Anatomia Animals, Facultat de Veterinàriaia, Universitat Autònoma de Barcelona (UAB), Campus de la UAB, 08193 Bellaterra, Catalonia Spain; 4 School of Public Health, Li Ka Shing, Faculty of Medicine, The University of Hong Kong, Hong Kong, China

**Keywords:** Air-liquid interface, alpaca, camelid, ex vivo model, MERS-CoV, tracheal explants

## Abstract

Middle East respiratory syndrome coronavirus (MERS-CoV) poses a serious threat to public health. Here, we established an ex vivo alpaca tracheal explant (ATE) model using an air-liquid interface culture system to gain insights into MERS-CoV infection in the camelid lower respiratory tract. ATE can be infected by MERS-CoV, being 10^3^ TCID_50_/mL the minimum viral dosage required to establish a productive infection. IFNs and antiviral ISGs were not induced in ATE cultures in response to MERS-CoV infection, strongly suggesting that ISGs expression observed in vivo is rather a consequence of the IFN induction occurring in the nasal mucosa of camelids.

**Supplementary Information:**

The online version contains supplementary material available at 10.1186/s13567-022-01084-3.

## Introduction, methods, and results

Middle East respiratory syndrome coronavirus (MERS-CoV) is the etiological agent causing a respiratory disease that emerged in The Kingdom of Saudi Arabia in 2012 [1]. Early reports from the Arabian Peninsula indicated high case-fatality rates associated with the disease [2–4]. As of April 2022, the World Health Organization has reported 2585 infections and 890 fatalities (∼34.4% case-fatality rate) globally in 27 countries across four continents [5]. The primary manifestation of MERS in humans ranges from asymptomatic or mild respiratory symptoms to pneumonia leading to acute respiratory distress syndrome [4]. Dromedary camels are reservoir/intermediate hosts of MERS‐CoV [6], since viral neutralizing antibodies have been reported in this species [7–13], and the virus does not require mutations to jump from dromedaries to humans [14]. Besides, all studied camelids are susceptible to MERS-CoV infection under both experimental and natural conditions [15–24].

Innate immunity, the first host defense system against viral infections, is thought to play a key role in MERS pathogenesis. Studies of MERS-CoV infections on numerous human cell types led to the conclusion that type I and III interferons (IFNs) are largely inhibited or delayed [25–27]. Furthermore, high and persistent secretions of inflammatory cytokines by lung macrophages and the inhibition of innate immune responses at the mucosal level are likely to contribute to a more severe infection in humans [28–31]. By contrast, camelids only show subclinical disease in response to MERS-CoV [15–24]. Such an outcome is apparently due to the action of type I and III IFNs generated by the infected nasal epithelia as suggested by recent work performed in alpacas [24]. Infected epithelium produced IFNs, unlike distant uninfected cells, while the expression of interferon-stimulated genes (ISGs) was elevated in both infected and uninfected epithelial cells, suggesting that IFNs could act in a paracrine fashion to induce ISGs expression in uninfected cells [24]. Similarly, IFNs produced at the nasal epithelium might also be sensed via an endocrine pathway, as evidenced by the production of ISGs in the lower respiratory tract (LRT) in absence of detectable expression of IFNs in these tissues [22, 24].

Besides the effective innate immune response of camelid hosts, another factor that may contribute to the mild pathogenesis of MERS-CoV in these species is the ability of the virus to moderately replicate within cells from the LRT. Previous research showed that very few infected cells were found in trachea upon intranasal MERS-CoV inoculation [15, 19, 20, 22, 24], although dipeptidyl peptidase 4 (DPP4) expression levels are comparable to that of nasal mucosa in camelids [32, 33]. It is unclear how MERS-CoV replication towards the LRT is restrained. Thus, in the present study, we established an ex vivo tracheal organ culture using an ALI system to fulfill these knowledge gaps.

**Figure 1:**
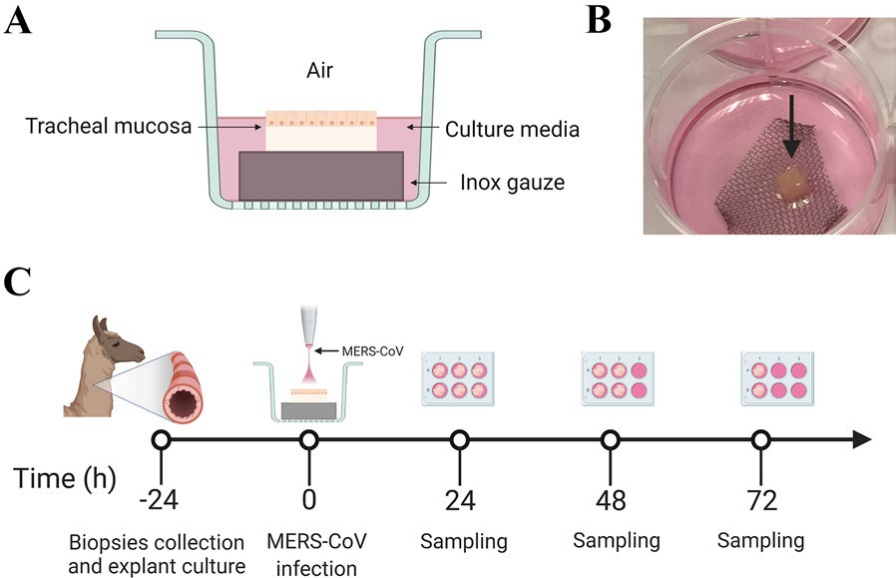
**Schematic diagram of the experimental time course and settings of the air-liquid (ALI) device for the alpaca tracheal explant (ATE) model.** (**A**) A schematic overview of the ALI interface set-up and (**B**) a photo of an ATE (arrow) resting onto an inox gauze within a 6-well culture plate. (**C**) General overview of the experiment indicating the time points in which explants were collected, cultured, infected, and sampled. Upon necropsy, ATE were isolated from alpacas (*n* = 3) and cultured in an ALI interface system. ATE were apically infected after 24 h of culture with MERS-CoV Qatar15/2015 strain at three different doses: 10^2^, 10^3^, and 10^4^ TCID_50_/mL. Explant replicates from each animal were collected for virological, pathological, and immunological assessments at -24, 0, 24, 48 and 72 h respectively.

Three 10 to 12-month-old alpacas (*Vicugna pacos*) were purchased by private sale, housed at IRTA farm facilities at Alcarràs (Catalonia, Spain) during the acclimation period and transferred to the Autonomous University of Barcelona, in Barcelona (Spain), for necropsy procedures. Animals were euthanized with an overdose of pentobarbital injected into the jugular vein. The middle portion of tracheas (*n* = 3) was aseptically collected and transported to the laboratory in pre-heated transport medium consisting of a 1:1 mixture of Dulbecco’s modified Eagle’s medium (DMEM, Lonza) and Roswell Park Memorial Institute (RPMI) 1640 medium supplemented with penicillin (100 U/mL), streptomycin (100 μg/mL), L-glutamine (10 mM); all of them from ThermoFisher Scientific (Life Technologies, Waltham, USA) and amphotericin (2 μg/mL; Sigma-Aldrich). Obtained tracheal tissues were washed by immersion into fresh warm transport medium three times, followed by the isolation of ATE according to a previously published protocol [34] with some modifications. Briefly, the tracheal mucosa was stripped from the tracheal rings. The cartilage was carefully discarded, and each segment was then cut into squares of 25 mm^2^, prior to transfer to fine meshed inox steel gauzes placed in 6-well culture plates with the epithelium facing up. Each well contained 2 mL of culture media (CM) consisting of transport media without amphotericin. ATE were cultivated in ALI interface, so the epithelium was slightly immersed in fluid with cilia exposed to the air (Figures 1A, B). Explants were cultured in a humidified incubator for up to 4 days at 37 °C and 5% CO_2_. The ciliary beating was checked daily (each 24 h) under a light microscope.

A passage-3 of the clade B MERS-CoV Qatar15/2015 strain (GenBank Accession MK280984) stock was propagated in Vero E6 cells and titrated by calculating the dilution that caused cytopathic effect in 50% of the inoculated cell cultures (50% tissue culture infectious dose endpoint [TCID_50_]), as previously described [19]. The MERS-CoV Qatar 15/2015 strain was selected on the basis that only clade B strains are currently circulating in the Arabian Peninsula [35] and it has enhanced in vivo replicative capabilities in alpacas compared to the prototypical clade A MERS-CoV EMC/2012 strain [22]. ATE were inoculated with the MERS-CoV Qatar15/2015 strain at three different doses: 10^2^, 10^3^, and 10^4^ TCID_50_/mL in CM (Figure 1C). Briefly, tracheal explants were prepared in triplicates for each time point per animal (*n* = 3) onto sterile stainless-steel gauzes placed on 6-well culture plates with the epithelial surface upwards (Figures 1A, B). After 24 h of culture, explants were then transferred to a new 6-well culture plate prior to infection with each MERS-CoV dose apically. CM alone was used for mock-infected controls. After 1 h of viral exposure in a humidified incubator at 37 °C and 5% CO_2_, explants were washed three times with warm PBS and placed back to the gauze at the original 6-well plates containing CM. Explants were collected at 24, 48 and 72 h post-inoculation (hpi) and introduced in tubes containing 1) 10% formalin for morphometric, histological and immunohistochemical analysis (two of the triplicates); or 2) TRI-Reagent (Zymo Research, California, USA) for RNA virus quantification as well as innate immune response analysis.

Explants were fixed by immersion in 10% neutral‐buffered formalin for 5 days, embedded in paraffin blocks and processed for histological analysis (hematoxylin and eosin stain, H&E). Besides, the thickness of the tracheal epithelium was assessed by measuring five randomly selected fields across each trachea section. A monoclonal mouse anti-MERS-CoV N protein antibody (Sino Biological Inc., Beijing, China) was used to detect the presence of MERS-CoV antigen, following a previously established protocol [33]. A grading system for immunohistochemistry (IHC) was established by a board-certified veterinary pathologist (−, no positive cells detected; ± , less than 10 positive cells per tissue section; + , 10–50 positive cells per tissue section; + + , 50 to 150 positive cells per tissue section).

Total RNA from ATE samples collected at different time points was extracted and converted into cDNA following a previous standard protocol [24]. A microfluidic RT-qPCR assay was utilized to relatively quantify the expression of immune related genes. The selection of innate immune genes and specific pairs of primers to amplify their transcriptional products were described in previous works [22, 24]. In addition, specific primers for the detection of genomic (UpE) and subgenomic (M) viral RNA were added to the assay (Sheet A in Additional file 1) [24]. Data were analyzed with the Real-Time PCR Analysis 4.1.3 software (Fluidigm Corporation, South San Francisco, USA) and the DAG expression software 1.0.5. 6, as previously described [24]. *HPRT1*, *GAPDH* and *UBC* genes were used as normalizer genes, and values obtained from infected explants collected at 24, 48 and 72 hpi were compared to those obtained from non-infected ATE at 0 hpi. The up-or down-regulated expression of each cytokine gene was expressed in fold-change values (Sheet B in Additional file 1).

**Figure 2:**
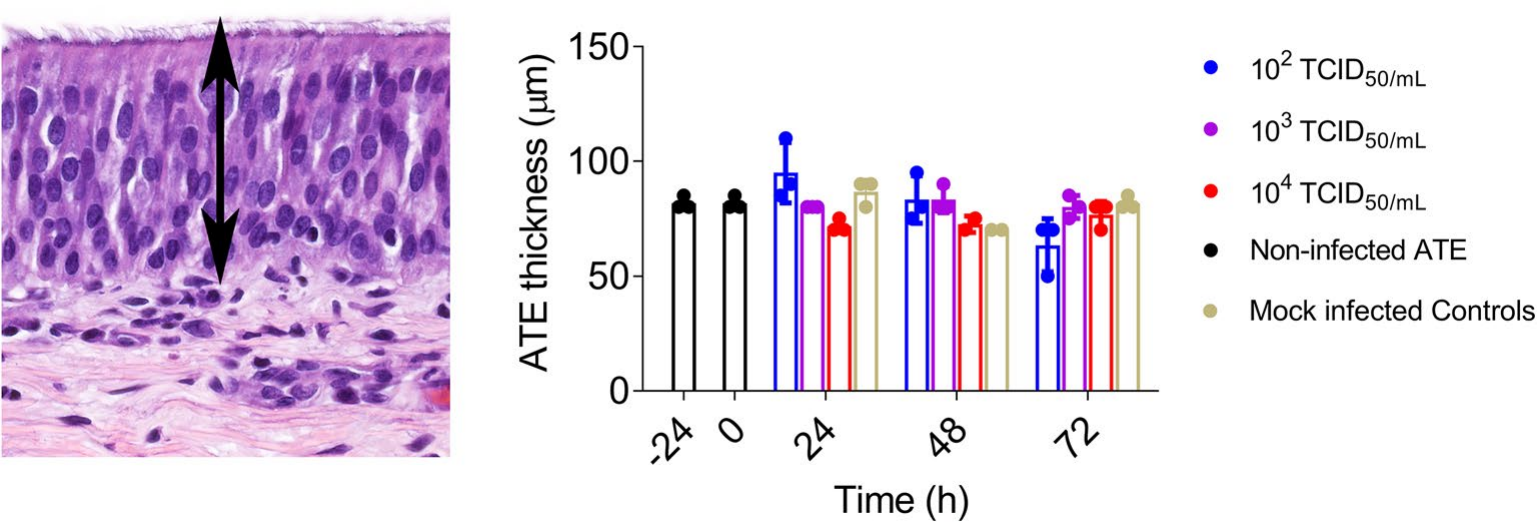
**Evaluation of alpaca tracheal explants (ATE) thickness after ex vivo culture by means of light photomicrographs.** The trachea was harvested upon necropsy, followed by infection at different doses of MERS-CoV (right of the figure). ATE were collected at different time points and were fixed in 10% neutral-buffered formalin. Sections of each formalin-fixed paraffin embedded ATE were stained by hematoxylin-eosin to evaluate the thickness of the epithelium by measuring five randomly selected fields across each section (indicated with a black arrow at the left) at –24, 0, 24, 48 and 72 hpi, respectively.

**Figure 3:**
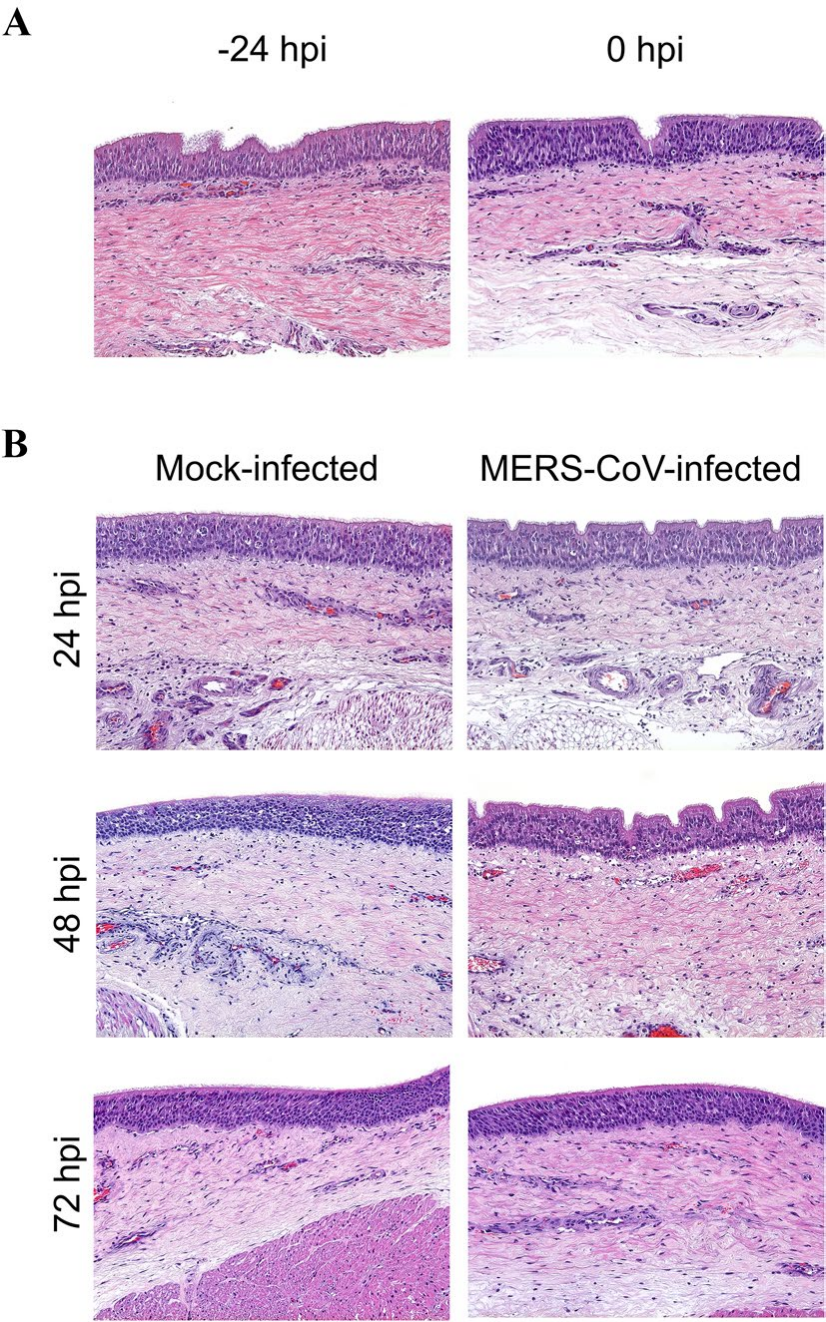
**Histopathological study of alpaca tracheal explants (ATE).** Hematoxylin-eosin staining was applied to assess histological alterations of ATE (**A**) prior and (**B**) after MERS-CoV infection. No significant lesions were observed in any of the studied explants, inoculated or not with MERS-CoV. Original magnification: × 200 for all tissues.

**Figure 4:**
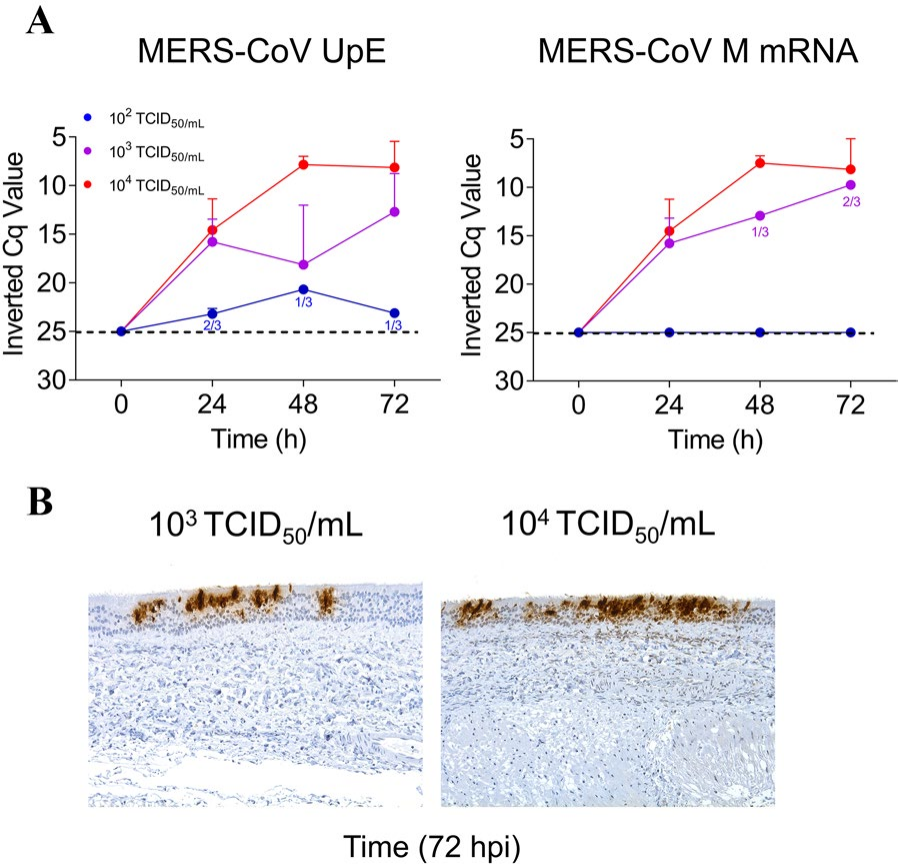
**MERS-CoV loads in alpaca tracheal explants (ATE).** (**A**) MERS-CoV genomic (left) and subgenomic (right) RNA was determined by microfluidic RT-qPCR assays (Sheet A in Additional file 1). Each line represents the mean Cq value and error bars display standard deviation intervals of ATE from 3 animals sampled at 0, 24, 48 and 72 hpi, respectively. Blue, purple, and red dots indicate ATE infected with 10^2^, 10^3^ and 10^4^ TCID_50_/mL MERS-CoV, respectively. (**B**) Tissue tropism of MERS-CoV in ATE. At 72 hpi, MERS-CoV N protein (brown staining) was detected by immunohistochemistry in the epithelium of ATE that were infected with 10^3^ and 10^4^ TCID_50_/mL doses respectively; original magnification: × 200 for both tissues. ATE, alpaca tracheal explants; 1/3 and 2/3 indicate the detection of viral RNA in one out of three or two out of three explants, respectively.

MERS-CoV infection did not significantly alter the average thickness of the epithelium layer (ranging between 63.3 to 86.7 µm) (Figure 2) nor caused obvious histopathological changes in ATE for the duration of the entire ex vivo experiment (Figures 3A, B). All cultured ATE maintained nearly intact cellular and tissular morphology despite vacuolation of very few cells within the epithelium and the lamina propria from 48 hpi onwards (Figure 3B). Figure 4A shows the kinetics of MERS-CoV yields after infection with three different viral doses (10^2^, 10^3^ and 10^4^ TCID_50_/mL, respectively). Viral genomic RNA loads in ATE were dose- and time-dependent (Figure 4A left). An infection dose of 10^4^ TCID_50_/mL led to the highest viral RNA detection in ATE and a plateau was reached at 48 hpi onwards. To ascertain active viral gene transcription within ATE, the presence of MERS-CoV subgenomic RNA (M gene) was also assessed (Figure 4A right). M mRNA loads were only detected in ATE infected with 10^3^ or 10^4^ TCID_50_/mL MERS-CoV, while no M mRNA transcription occurred upon inoculation with 10^2^ TCID_50_/mL doses. In agreement with the results obtained by viral RNA quantification, IHC positive labeling was detected in ATE infected with either 10^3^ or 10^4^ TCID_50_/mL sampled at 48 or 72 hpi (Figure 4A and Additional file 2). Specific staining was mostly located in the cytoplasm of tracheal epithelial cells (Figure 4B).

**Figure 5:**
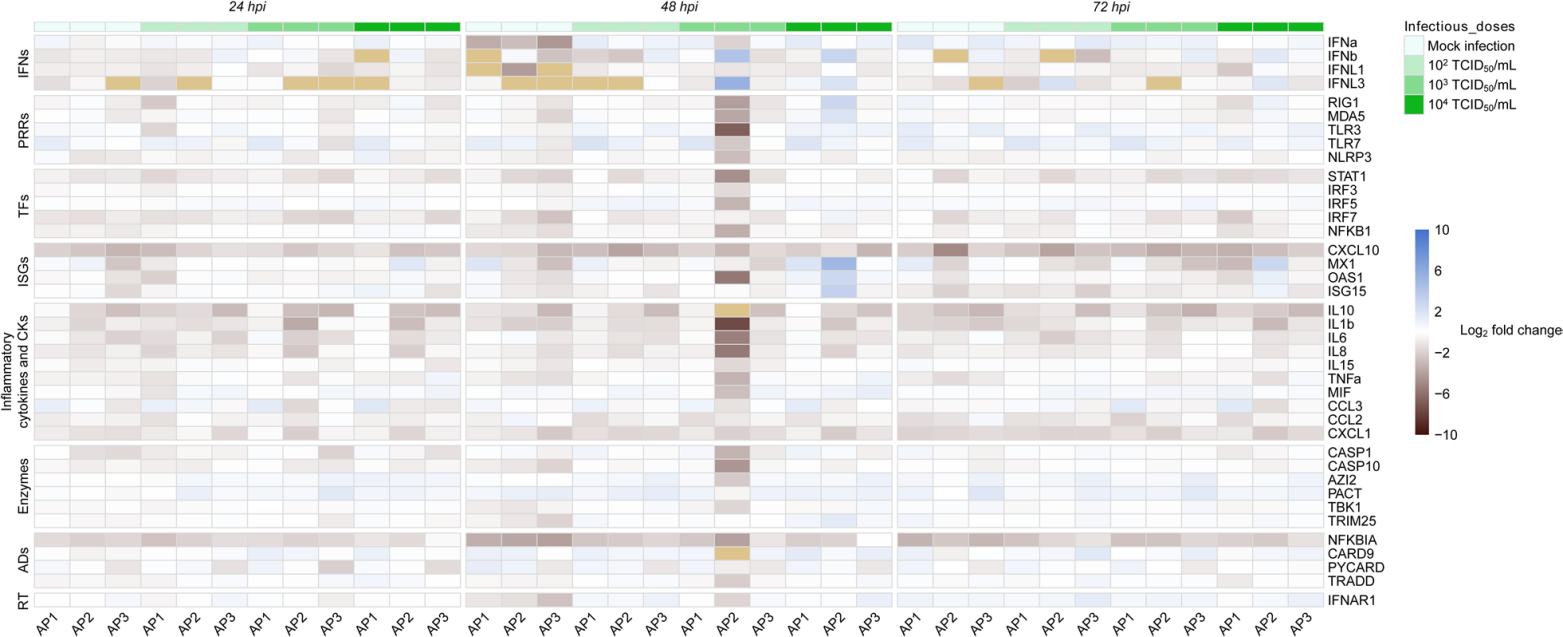
**Kinetics of innate immune gene profiles of ATE in response to different MERS-CoV infectious doses.** Total RNA was extracted from ATE, followed by conversion to cDNA. The Fluidigm Biomark microfluidic assay was used to quantify transcripts from innate immune genes at different hpi. After normalization, fold change values between controls (freshly prepared explants) and mock or infected ATE were calculated. The resulting heatmap shows color variations corresponding to log_2_ fold change values, blue for increased and brown for decreased gene expression, respectively. The khaki rectangles indicate no expression of the corresponding gene. TFs, transcription factors; CKs, chemokines; ADs, adaptors; RT, receptor.

To explore antiviral and inflammatory pathways activated upon MERS-CoV infection in ATE, the relative mRNA expression levels of 39 innate immune response genes were monitored in mock-treated and viral-infected ATE. Three highly stable normalizer genes were included in the assay. All genes were detected at basal levels in non-infected ATE controls collected at 0 hpi, thus, allowing normalization of the whole data sets against this reference time point. Transcription of pro-inflammatory cytokines, chemokines, and transcription factors (except for IRF5 that remained unaltered) showed a slight down-regulation trend upon infection of ATE with any of the MERS-CoV dosages (Figure 5 and Sheet B in Additional file 1). The rest of the genes, including IFNs, ISGs, pattern recognition receptors, downstream signaling enzymes, adaptors, and receptors, fluctuated around basal levels (Figure 5 and Sheet B in Additional file 1), suggesting inhibition of antiviral responses in ATE upon MERS-CoV infection.

## Discussion

The pathogenesis of MERS-CoV in the intermediate host is yet to be fully understood. Although camelids can be experimentally infected, such an approach represents a significant financial cost, ethical concern, safety risk and requires special household facilities under a biosafety level-3 (BSL-3) environment. Thus, we developed an ex vivo ATE model to address these hurdles. ATE retained the ciliary integrity during the course of the experiment, indicating that the coordination and modulation of the ciliary beating of tracheal epithelium remained functional. Morphometric and histological assessments demonstrated that ATE remained integral with good cellular and tissular morphology for at least 72 hpi, showing great resemblance to the freshly collected trachea. Of note, a massive ciliary loss occurs in the nasal epithelia of dromedary camels experimentally infected with MERS-CoV [36] but not in llamas [33] or alpacas [24].

In the present study, ATE were unable to express IFNs in agreement with previous observations on the trachea of alpacas infected with MERS-CoV [22, 24]. In addition, ISGs were not induced in ATE upon MERS-CoV infection, supporting the fact that upregulation of ISGs in tracheal mucosa observed in vivo is due likely to the paracrine action of IFNs produced by the nasal epithelia [24]. Recently, pseudostratified airway epithelial cell (AEC) culture models for llamas and Bactrian camels have been developed. These AEC were generated from the tracheobronchial tract, supported the growth of several MERS-CoV lineages and were sensitive to the antiviral effect of IFNs [37, 38]. However, in these experiments, the production of IFNs and cytokines upon MERS-CoV infection were not checked. Nevertheless, when treated with IFNs, camelid tracheal cells can produce ISGs, denoting a functional innate immune system [38]. Both, ATE and AEC could be exploited to test agonists and antagonists of several innate immune pathways leading to MERS-CoV clearance.

The exclusive use of camelid tracheal cells to study host-MERS-CoV interactions might not be sufficient since, at least in vivo, infected nasal epithelial cells are able to notably up regulate the transcriptional expression of IFN genes [22, 24]. Therefore, the development of nasal explants and well differentiated nasal epithelial cell models will be necessary for future studies to fully appreciate the interplay between the epithelial barrier and MERS-CoV infection in camelids. In that respect, explant models might better reflect an in vivo scenario since mucosa and submucosa are both co-cultured maintaining the tissue structure. Nonetheless, AEC and tracheal explant models overcome natural physiological drawbacks inherent to epithelial cell lines including differences in cell polarization, coordinated ciliary activity and apical contact with air [39]. Furthermore, tissue pieces obtained from a single animal can be used in numerous replicates, thereby reducing the experimental variability and the number of animals used, in line with the 3Rs principle [40].

In conclusion, a novel ex vivo culture model was developed using ATE, which is suitable to study MERS-CoV infection and replication as it occurs in natural reservoir hosts. We demonstrated as a proof of principle that ATE can be infected by MERS-CoV without triggering innate antiviral responses. Besides, ATE could also be suitable to study other respiratory pathogens of camelids by reducing the burden of animals used for experimentation.

## Supplementary Information

**Additional file 1:** **Viral loads (UpE and M mRNA) and Fc of innate immune response genes in ATE**.

**Additional file 2:** **MERS-CoV N protein detection by IHC in ATE**.

## Data Availability

The data that support the findings of this study are available from the authors upon reasonable request.

## Abbreviations

AEC: pseudostratified airway epithelial cell
ATE: alpaca tracheal explant
BSL-3: biosafety level-3
CM: culture media
DPP4: dipeptidyl peptidase 4
hpi: hours post-inoculation
IFN: interferon
IHC: immunohistochemistry
ISG: interferon-stimulated genes
LRT: lower respiratory tract
MERS-CoV: Middle East respiratory syndrome coronavirus
TCID_50_: 50% tissue culture infectious dose endpoint
