# Supplementary material for: Evaluation of alpaca tracheal explants as an ex vivo model for the study of Middle East respiratory syndrome coronavirus (MERS-CoV) infection
Source: Vet Res. 2022 Sep 2;53:67. doi: 10.1186/s13567-022-01084-3 (PMC9438371; doi:10.1186/s13567-022-01084-3)
Supplement: Supplementary file 2 — Additional file 2: MERS-CoV N protein detection by IHC in ATE. [file 13567_2022_1084_MOESM2_ESM.docx]

**Additional file 2 MERS-CoV N protein detection by IHC in ATE**

|  | 24 hpi  AP3 | | 48 hpi | | 72 hpi | |
| --- | --- | --- | --- | --- | --- | --- |
|  | IHC | No. | IHC | No. | IHC | No. |
| Mock infection | － | 0/3 | － | 0/3 | － | 0/3 |
| 10^2^ TCID_50_/mL | － | 0/3 | － | 0/3 | － | 0/3 |
| 10^3^ TCID_50_/mL | － | 0/3 | － | 0/3 | + | 2/3 |
| 10^4^ TCID_50_/mL | － | 0/3 | ++ | 1/3 | ++ | 2/3 |

Abbreviations: IHC, immunohistochemistry; No., number of the animals with MERS-CoV labelled cells present in their respective ATE; hpi, hours post-infection; -, no positive cells detected; +/-, less than 10 positive cells per tissue section; +, 10 to 50 positive cells per tissue section; ++, 50 to 150 positive cells per tissue section.
